# Systematic Review of Randomized Controlled Trials of Different Types of Patch Materials during Carotid Endarterectomy

**DOI:** 10.1371/journal.pone.0055050

**Published:** 2013-01-31

**Authors:** Shiyan Ren, Xianlun Li, Jianyan Wen, Wenjian Zhang, Peng Liu

**Affiliations:** 1 Cardiovascular Center, China-Japan Friendship Hospital, Beijing, People’s Republic of China; 2 Clinical Research Institute, China-Japan Friendship Hospital, Beijing, People’s Republic of China; University of Leicester, United Kingdom

## Abstract

**Background and Purpose:**

Carotid endarterectomy (CEA) with patch angioplasty produces greater results than with primary closure; however, there remains uncertainty on the optimal patch material in CEA. A systematic review of randomized controlled trials (RCTs) was performed to evaluate the effect of angioplasty using venous patch versus synthetic patch material, and Dacron patch versus polytetrafluoroethelene (PTFE) patch material during CEA.

**Methods:**

A multiple electronic health database screening was performed including the Cochrane library, Pubmed, Ovid, EMBASE and Google Scholar on all randomized controlled trials (RCTs) published before November 2012 that compared the outcomes of patients undergoing CEA with venous patch versus synthetic patch. RCTs were included if they compared carotid patch angioplasty with autologus venous patch versus synthetic patch material, or compared one type of synthetic patch with another.

**Results:**

Thirteen RCTs were identified. Ten trials, involving 1946 CEAs, compared venous patch with synthetic patch materials. Two trials, involving 400 CEAs in 380 patients, compared Dacron patch with PTFE patch. The hemostasis time in CEA with PTFE patch was significantly longer than with venous patch (*P*<0.0001), and longer than with Dacron patch (*P*<0.0001). There was no significant difference of mortality rate, stroke rate, restenosis, and operative time in CEA with venous patch versus synthetic patch material, or in CEA with Dacron patch versus PTFE patch (all *P*>0.05). One RCT of 95 CEAs in 92 patients compared bovine pericardium with Dacron patch, and demonstrated a statistically significant decrease in intraoperative suture line bleeding with bovine pericardium compared with Dacron patch (*P*<0.001).

**Conclusions:**

The hemostasis time in CEA with PTFE patch was longer than with venous patch or Dacron patch. The overall perioperative and long-term mortality rate, stroke rate, restenosis, and operative time were similar when using venous patch versus synthetic patch material or Dacron patch versus PTFE patch material during CEA. More data are required to clarify differences between different patch materials.

## Introduction

Carotid endarterectomy (CEA) has been considered as one of the important procedures to treat patients with severe stenosis of carotid artery. However, patients may have postoperative restenosis of carotid artery [1] and subsequent recurrent ipsilateral ischemic stroke in high-grade recurrent stenosis. One solution to these problems is patch angioplasty in CEA [2], [3]. Several systematic reviews have compared the results of the primary closure of arteriotomy with routine patch closure during CEA [4]–[6], and the outcomes favor patch angioplasty over primary closure in reducing risk of stroke and restenosis [4]–[6]. However, there remain reports that the difference was insignificant and that there was no benefit from the routine use of patch angioplasty in CEA [7].

A variety of patch materials for closure of the arteriotomy are available, including autologous venous patch and synthetic patch materials (Dacron, polytetrafluoroethelene (PTFE), bovine pericardium, and polyester urethane) [4]–[8]. Currently, selection of types of patch materials depends on the surgeon’s preference, as there is no agreement on the priority of use of venous over synthetic patch materials during CEA [1]. Moreover, all randomized controlled trials (RCTs) on this issue so far have been underpowered because the number of patients involved was not large and the studies were unblinded. However, better evidence is not yet available; therefore, the aim of this paper is to update the review of RCTs via a meta-analysis to compare venous patch with synthetic patch materials, and different synthetic patch materials during CEA.

## Materials and Methods

### Literature Search

The authors screened and identified various databases, including the Cochrane library, Pubmed, Ovid,Embase, and Google scholar, before November 2012. The following key words were used: “carotid artery stenosis, endarterectomy with venous patch or synthetic patch, saphenous vein patch, or jugular vein patch; and patch angioplasty”. The reference lists of reviews and retrieved papers were searched manually. Language was not restricted in the literature search.

### Inclusion Criteria and Exclusion Criteria

All RCTs papers that compared autologous venous patch versus synthetic patch material, or different types of synthetic patch during CEA were included. Exclusion criteria were RCTs comparing patch angioplasty with primary closure during CEA, non-RCT studies, abstracts or unpublished reports, case reports, and reviews.

### Data Extraction

Titles and abstracts of all citations and searched papers were initially screened, and the eligible full-text articles were obtained. Two independent reviewers (Ren S, Li X) screened, selected, and cross-checked all the eligible papers, and discussed the disagreements on the eligibility of included papers in order to reach an agreement. In each trial, the number of patients and all the outcomes of treatment were identified. A greater than 50% restenosis or occlusion of the operated artery was defined by duplex ultrasound scan, or angiography.

The methodological quality of RCTs was assessed using the Jadad studies method [9], and any publication bias was assessed using funnel plots.

### Statistical Analysis

Statistical analysis of categorical variables were performed using risk ratio (RR) as a summary statistic, mean differences were used for analysis of continuous data. An RR<1 favors the experimental (venous) patch group or the Dacron patch. The Software Review Manager (RevMan 5.1.7, Cochrane Collaboration, Oxford, UK) was used for statistical analysis. Heterogeneity among the RCTs results was evaluated with the standard Chi-square test to determine whether to use the fixed- or random-effects model. The Mantel-Haenszel method was used to combine the RR for the results of interest using a random-effects meta-analytical technique. *P* value less than 0.05 was considered statistically significant.

## Results

**Figure 1:**
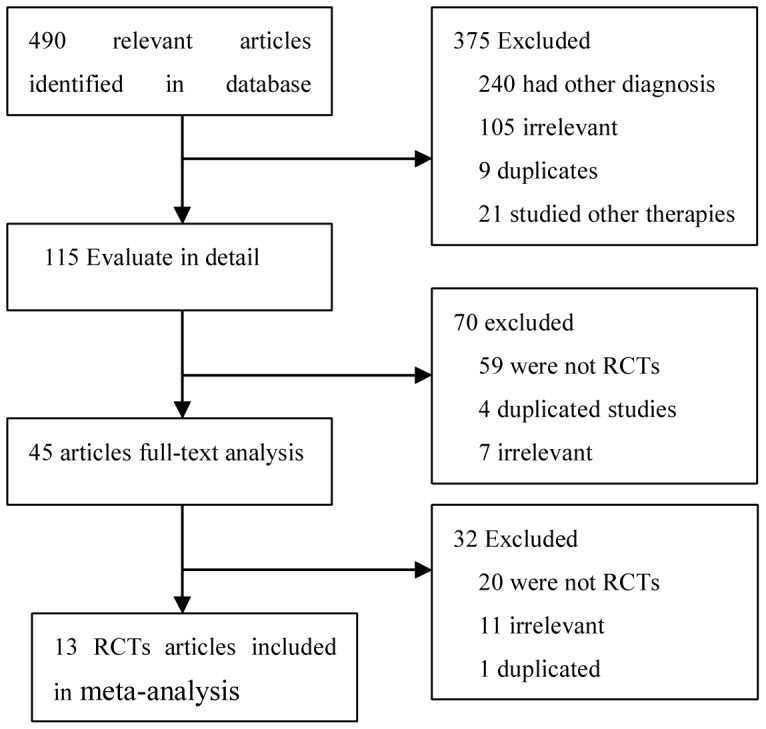
Flow diagram showing different steps of the systematic review. *RCTs:* randomized controlled trials.

The significant complications after CEA included bleeding from or rupture of the patched artery, reoperation, wound infection, and wound hematoma. Initially, 490 papers were searched through the keyword search, and 445 papers were excluded after further reviewing the title and abstracts of the papers. The remaining 45 papers were carefully reviewed, and 14 articles conforming to the eligibility criteria were included in this study (Fig. 1, Table 1) [10]–[23]. Three of 14 articles compared results of Dacron patch with PTFE during CEA, of which two articles were the same RCT reporting the early and follow-up outcomes in different journals, thus these three studies had a subtotal of 380 patients who underwent 400 CEAs (Table 1). Ten of the 14 studies selected compared outcomes of autologus venous patch with synthetic patch materials during CEA, and contained a combined total of 1909 subjects, of whom 1946 CEAs were performed. One RCT compared the outcomes of CEA using bovine pericardium with Dacron [10].

**Table 1:** Details of randomized controlled trials.

Trials	Year	No. of patients (no.operations)	Mean age(y)	Sex (% male)	Venous or synthetic patch	Syntheticpatch type	FU time
Marien BJ [10]	2002	92 (95)	66	64.2	Dacron	BP	Perioperative
Grego F [11]	1996	160 (160)	70	72.5	EJV	PTFE	mean 4 y
O’Hara PJ [12]	1996	195 (207)	69	73.6	ASV	Dacron	18 mon
Hayes PD [13]	2001	274 (276)	70.5	66.3	SV	Dacron	30 days
AbuRahma AF [14]	1996	399 (357)	68	53	VPC (ankle)	PTFE	mean 30 mon
Lord RS [15]	1989	123 (140)	63	62	SV	PTFE	12 mon
Gonzalez-fajard JA [16]	1994	84 (95)	69.5	88.1	SV	PTFE	29 mon
Ricco JB [17]	1994	124 (141)	63	80	SV	PTFE	mean 53 mon
Katz SG [18]	1996	190 (207)	72	49.3	SV (thigh)	Dacron	Not mention
Naylor R [19]	2004	273 (276)	71	67	SV	Dacron	3 y
Meerwaldt R [20]	2008	87 (87)	67	79.6	SV (ankle)	Fluoropassiv	24 mon
AbuRahma AF [21]	2002	180 (200)	68.3	53	Dacron	PTFE	30 days
AbuRahma AF [22]	2003	180 (200)	68.3	53	Dacron	PTFE	36 months
AbuRahma AF [23]	2007	200 (200)	68	49.5	Dacron	PTFE	Perioperative

ASV ankle saphenous vein, *BP* bovine pericardium, *EJV* external jugular vein, *FU* follow up, *PTFE* polytetrafluoroethylene patch, *SV* saphenous vein, *VPC* vein patch closure, *Y* year, *Mon* month.

Table 1 shows the main outcomes and characteristics of each study. Two trials had three arms: primary closure, venous patch, and PTFE patch [15], [17]. For the analysis of patients with bilateral carotid artery stenosis, the first CEA and contralateral CEA were counted (Table 1).

### Trials of Venous Patch versus Synthetic Patch Materials during CEA

**Figure 2:**
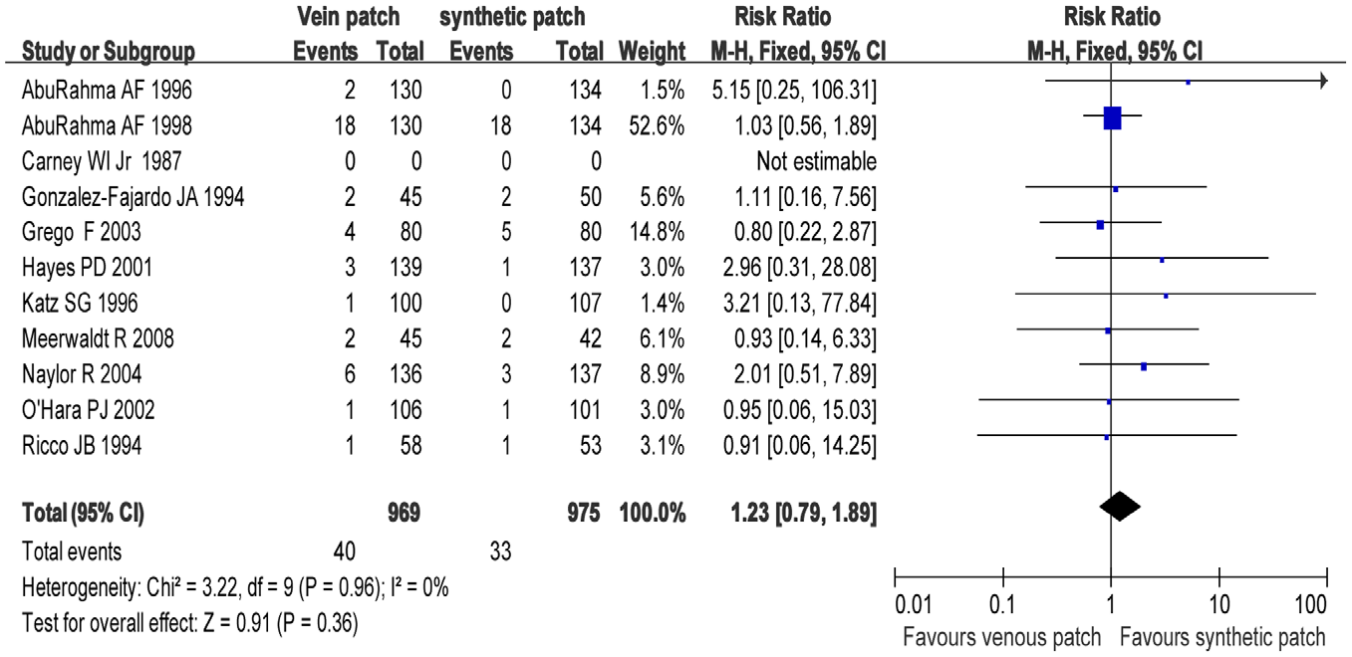
Mortality in both groups. Graphical representation of the results. *M-H* : Mantel-Haenszel.

**Figure 3:**
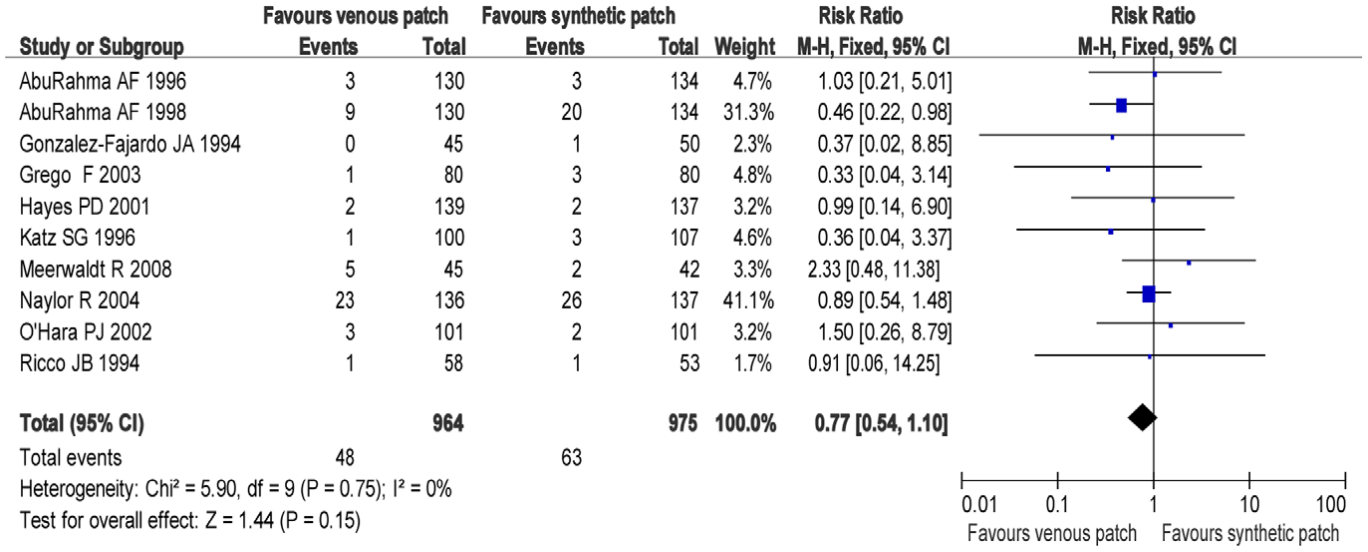
Any stroke event is compared in both groups. Graphical representation of the results. *M-H:* Mantel-Haenszel.

**Figure 4:**
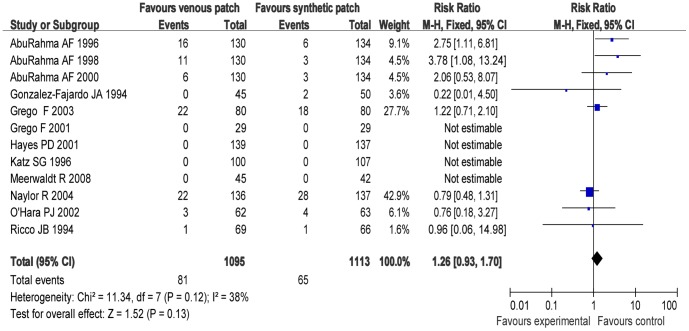
Restenosis of carotid artery in both groups. Graphical representation of the results. *M-H:* Mantel-Haenszel.

**Figure 5:**
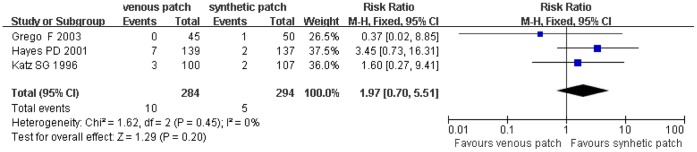
Postoperative wound infection events in both groups. Graphical representation of the results. *M-H* : Mantel-Haenszel.

**Figure 6:**
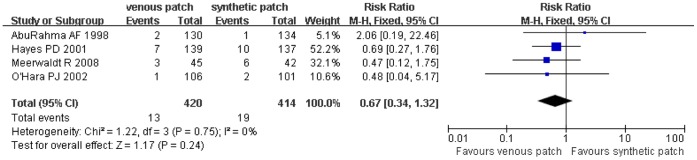
Reoperation for wound hematoma compared in both groups. Graphical representation of the results. *M-H:* Mantel-Haenszel.

**Figure 7:**
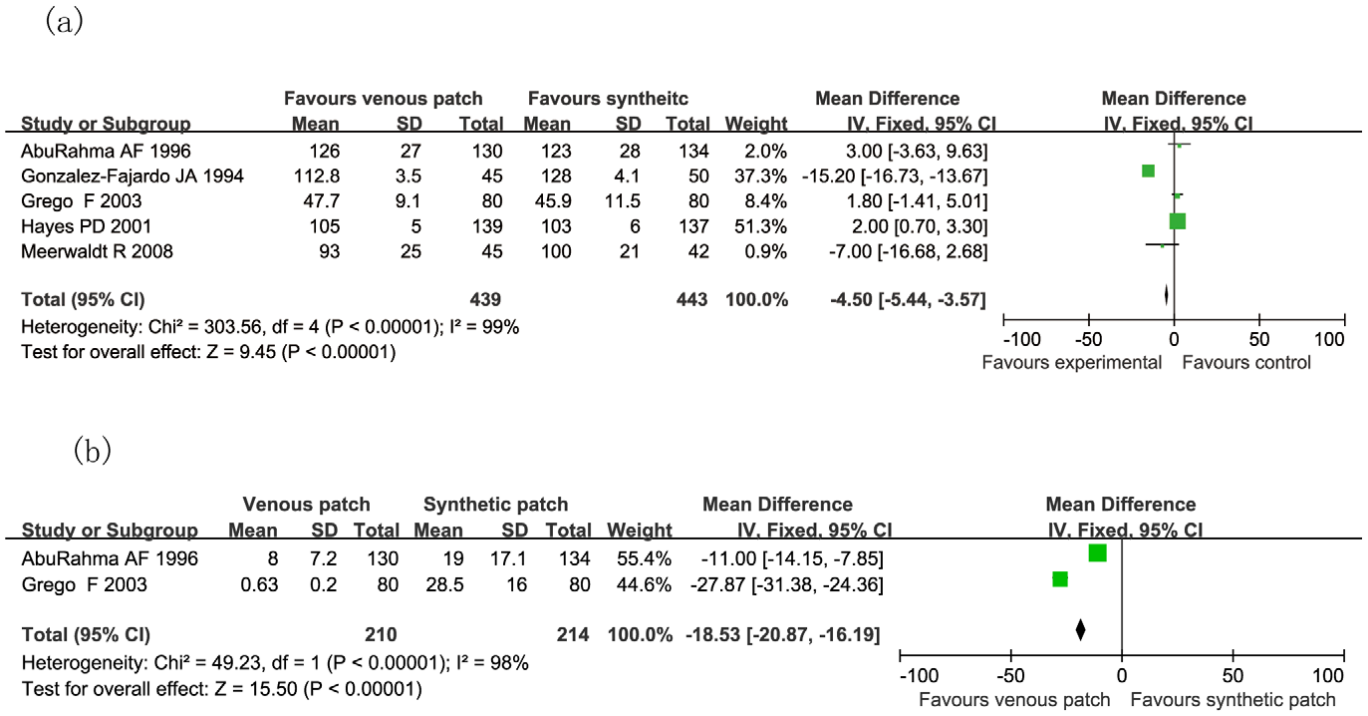
Mean operative time (a) and mean hemostasis time (b) in minutes are compared in both groups. Graphical representation of the results.

Figures 2–7 are the forest plots showing the outcomes of meta-analysis of the outcomes of CEA with venous patch versus synthetic patch material. There was no significant difference between CEA with venous patch versus synthetic patch material in the incidence of mortality (RR: 1.23; 95% CI: 0.79, 1.89; *P* = 0.36; Fig. 2), any stroke events (RR: 0.77; 95% CI: 0.54, 1.10; *P* = 0.15; Fig. 3), or restenosis of carotid artery (RR: 1.26; 95% CI: 0.93, 1.70; *P* = 0.13; Fig. 4). Similarly, no significant difference between the two groups was observed in terms of incidence of postoperative wound infection (RR: 1.97; 95% CI: 0.70, 5.51; *P* = 0.20; Fig. 5), incidence of reoperation for wound hematoma (RR: 0.67; 95% CI: 0.34, 1.32; *P* = 0.24; Fig. 6); however, mean operative time (Mean difference: −0.45; 95% CI: −5.44, −3.57; *P*<0.00001; Fig. 7a), and the hemostasis time (Mean difference: −18.53; 95% CI: −20.87, −16.19; *P*<0.00001; Fig. 7b) in the synthetic patch group was significantly longer than in venous patch group.

### Outcomes of RCTs Comparing Dacron Patch with PTFE

**Figure 8:**
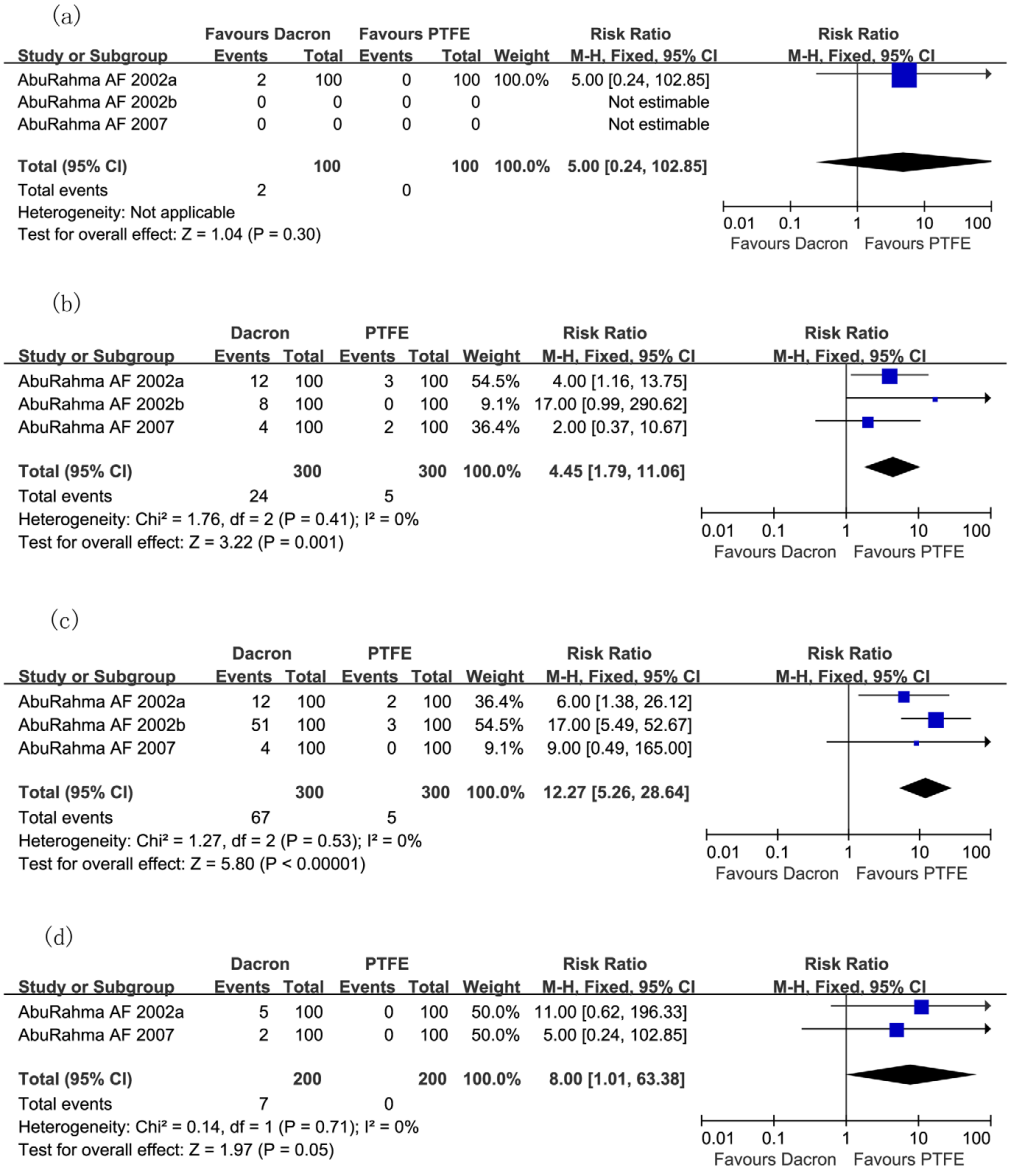
Meta-analysis of incidence of mortality rate (a), TIA and stroke (b), 50% restenosis to occlusion of carotid artery (c), and carotid thrombosis (d) after carotid endarterectomy, comparing Dacron and PTFE during CEA in randomized controlled trials. *CEA* carotid endarterectomy; *M-H* Mantel-Haenszel; *PTFE* polytetrafluoroethelene.

**Figure 9:**
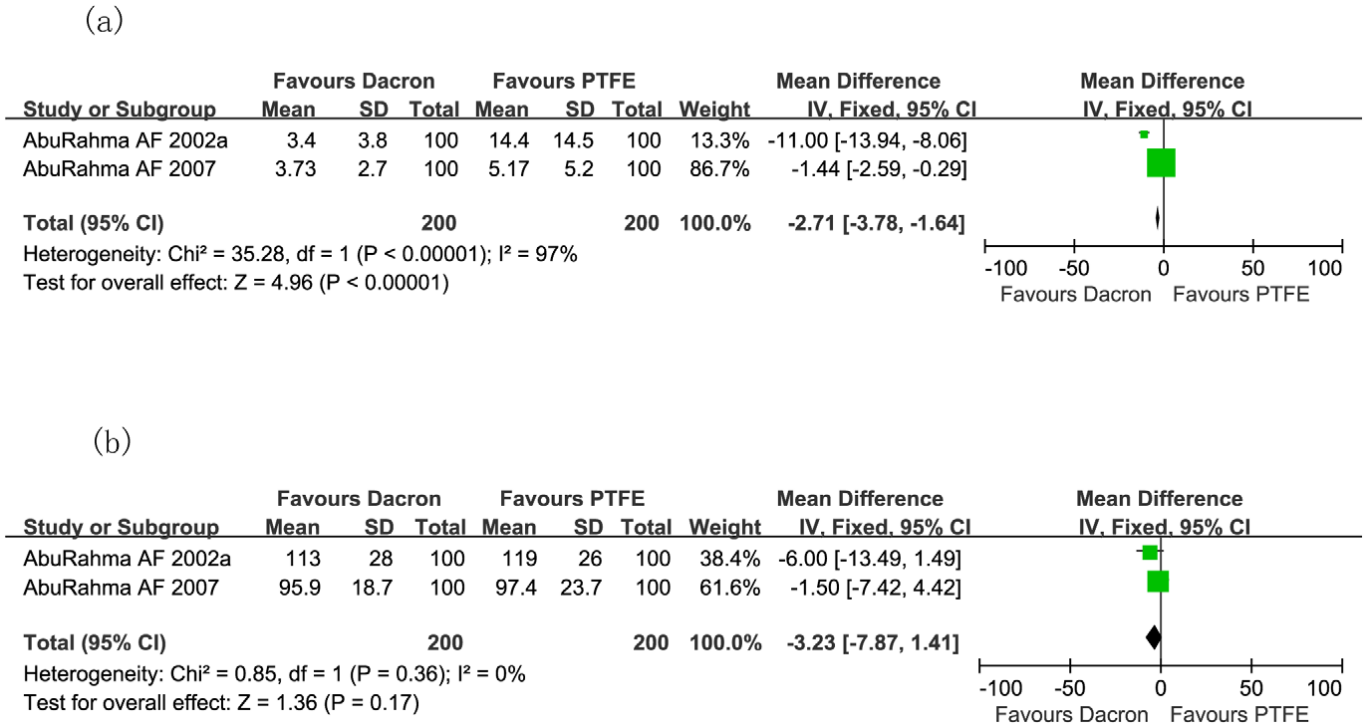
Meta-analysis of hemostasis time (a), and operative time (b) in minutes during carotid endarterectomy, comparing Dacron and PTFE during CEA in two randomized controlled trials. *CEA:* carotid endarterectomy; *PTFE:* polytetrafluoroethelene.

Fig. 8 demonstrates the incidence of transient ischemic attack (TIA) and stroke (RR: 4.45; 95% CI: 1.79, 11.06; *P* = 0.001; Fig. 8b), 50% restenosis to occlusion of carotid artery (RR: 12.27; 95% CI: 5.26, 28.64; *P*<0.00001; Fig. 8c), and carotid thrombosis (RR: 8.00; 95% CI: 1.01, 63.38; *P* = 0.05; Fig. 8d) after CEA were significantly higher in the Dacron patch group than in PTFE patch group, although incidence of mortality rate did not differ significantly (RR: 5.00; 95% CI: 0.24, 102.85; *P* = 0.30; Fig. 8a). However, the hemostasis time in the PTFE patch cohort was significantly longer than in Dacron patch cohort (Mean difference: −2.71; 95% CI: −3.78, −1.64; *P*<0.00001; Fig. 9a), even though the operative times between both groups were similar (Mean difference: −3.23; 95% CI: −7.87, 1.41; *P* = 0.17; Fig. 9b).

### Results of RCT Comparing Bovine Pericardium with Dacron

One RCT [10] of 95 CEAs in 92 patients comparing bovine pericardium with Dacron patch observed bleeding at 3 and 4 minutes after removal of the carotid cross-clamp, and then objectively weighed the sponge used to tamponade bleeding during these time intervals. The incidence of suture line bleeding at 3 minutes was 14% (7/51) in the bovine pericardium group and 55% (24/44) in the Dacron group (*P*<0.001). Suture line bleeding at 4 minutes was present in 4% (2/51) in the bovine pericardium group and 30% (13/44) in the Dacron group (*P* = 0.001). Weight of total intraoperative suture line bleeding (Net±SEM sponge weight) in the bovine pericardium group was significantly less than in Dacron group (6.25±0.55 g versus 16.34±1.85 g; *P*<0.001).

### Methodological Quality of Included Studies

The study quality was assessed on the methods described by Jadad for randomized studies [9]. The randomization sequence in most trials was well concealed using sealed, opaque, sequentially-numbered envelopes. However, there were significant flaws in some trials, as no detailed randomization method was reported. Blinding is important in reducing bias in the detection of some operative results, yet the detailed blinding method was not mentioned in the reports.

The events of stroke and death were too few to determine whether there were significant differences between venous patch and synthetic patch during either the perioperative period or follow-up; thus, the results of trials were compared at the end of follow-up. One trial provided a definition of peudoaneurysm, but no ruptured pseudoaneurysm or related stroke was reported.

### Publication Bias

Funnel plots were performed to test if publication bias existed within the studies included in the meta-analysis, none of the papers laid outside the limits of the 95% CI (Funnel plots were not shown).

## Discussion

Several studies have showed that patch angioplasty is better than primary closure in CEA in lowering the risk of restenosis of carotid artery and stroke [2], [3], [5], [24]. However, there is no consensus on the optimal patch material, and the available data do not support the use of venous patch over synthetic patch materials during CEA [4]–[6]. The present meta-analysis results indicate that the outcomes of CEA with venous patching was similar to that with synthetic patching in terms of reducing risks of stroke or death, and recurrent stenosis during the perioperative period and long-term follow-up, but the hemostasis time in CEA with synthetic patch was significantly longer than in CEA with venous patch material. Due to the different types of synthetic patches used in the present study, and that different synthetic patch materials act variably, we further compared the outcomes of CEA using Dacron and PTFE materials. The data show that incidences of TIA and stroke, restenosis (from 50% to occlusion) of carotid artery, and carotid thrombosis after CEA were significantly higher in Dacron patch group than in PTFE patch group (*P*<0.05), but the mortality rate was similar in both groups (*P* = 0.3), and the hemostasis time in the PTFE group was significantly longer than in the Dacron group. Furthermore, bovine pericardium is superior to Dacron in reducing intraoperative suture line bleeding (*P*<0.001).

The benefit of patch angioplasty in CEA is clear in patients with narrow arteries [25]. Carotid patching plays a role in reducing risk of stroke, especially in the carotid artery with a narrow internal lumen or a long plaque [25]. However, there is no clear agreement on the size of the artery lumen required for patch angioplasty. Few authors have reported the size of internal carotid artery in RCTs [25]. However, it is generally accepted that a patch angioplasty is indicated for internal carotid artery diameter <4–5 mm to prevent perioperative stroke rates and occlusion, and [25].

There remains controversy on the choice of patch materials in CEA. Selection of patch material is affected by thrombogenicity, aneurismal formation, risk of patch rupture, availability of patch material, complications related to vein harvesting, and the resistance to infection. Some surgeons prefer harvesting autologous veins, including the saphenous vein, or the internal/external jugular vein and facial vein [8]. RCTs and animal studies support that using an intima-lined patch may potentially reduce the risk of perioperative thrombosis and infection [26]. Indeed, vein-patch walls did not develop a thickened intima [26]. However, complications with saphenous vein patch following CEA have been reported, including a longer operating time, a blow-out or patch rupture, potential risk of false aneurysm formation, thrombosis from dilated or aneurismal carotid dilation [4], [27]–[29] in the postoperative period, and restenosis on long-term follow-up [30], [31].

The benefits of synthetic patches, including the Dacron and PTFE, are easy availability, resistance to aneurismal formation and patch rupture, lack of morbidity caused by vein harvesting, and preservation of vein conduits intact available for future potential coronary artery bypass grafting. However, it has been reported that Dacron synthetic patch is at risk of infection and thrombogenicity after CEA [18] and the PTFE patch causes a prolonged bleeding in CEA [18]. Our meta-analysis showed that the mean hemostasis time for the PTFE patch was significantly higher than for venous patch. We further compared the Dacron patch with the PTFE patch materials during CEA, and the results showed that the hemostasis time was still longer in CEA using PTFE patch than Dacron patch. Even using the new type of PTFE (Gore-Tex® Acuseal, W.L Fore & Associates Inc., Newark, USA), one RCT trial showed that hemostasis time in PTFE was longer than in Dacron patch group (*P* = 0.01) [23]. Similarly, excessive intraoperative bleeding from needle holes in the conventional PTFE patch was reported in earlier studies [27], [32]. Reduction of such blood loss has been found to be associated with a needle/suture diameter ratio of 1∶1 [27]. It is reported that use of PTFE suture, CV-6 (Gore-Tex® Acuseal) and polypropylene sutures (prolene 5/0) with RB-1 needles (TT-9) could minimize hemostasis time [23], [27]. Therefore, the hemostasis issue should be considered for the selection of the patching materials. In addition, the surgeon may prefer venous patching in the event that patients refuse to use the costly synthetic patch for CEA.

Limitations of this study include heterogeneity of synthetic patches, variety of follow-up periods, and statistically underpowered number of patients in each trial. Thus, these results may not be completely reliable and should be interpreted cautiously. In addition, a new type patch of PTFE (Gore-Tex® Acuseal) is reported to have a greater outcome than the conventional PTFE patch [23], and a new collagen-impregnated Dacron patch has been designed to restrict its thrombogenicity.

### Conclusions

The hemostasis time in CEA with PTFE patch is longer than with venous patch or Dacron patch. The overall perioperative and long-term mortality rate, stroke rate, restenosis, and operative time are similar when using venous patch versus synthetic patch, or using Dacron patch versus PTFE patch during CEA. Nevertheless, larger cohorts of patients are warranted to demonstrate the optimal patch materials, and the priority of autologus venous patch versus synthetic patch.

## Supporting Information

Table S1 PRISMA flow diagram of the meta-analysis.
(DOC)

Table S2 PRISMA checklist of the meta-analysis.
(DOC)
