# Supplementary material for: Systematic Review of Randomized Controlled Trials of Different Types of Patch Materials during Carotid Endarterectomy
Source: PLoS One. 2013 Jan 31;8(1):e55050. doi: 10.1371/journal.pone.0055050 (PMC3561447; doi:10.1371/journal.pone.0055050)
Supplement: Table S1 — PRISMA flow diagram of the meta-analysis. (DOC) [file pone.0055050.s001.doc]

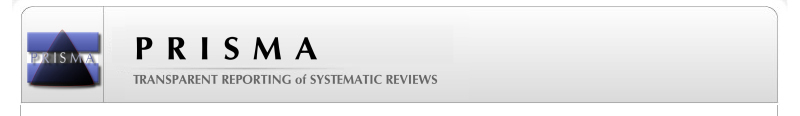
**PRISMA 2009 Flow Diagram**

**Screening**

**Included**

**Eligibility**

**Identification**

Records identified through database searching
(n =293 )

Additional records identified through other sources
(n =197 )

Records after duplicates removed
(n = 115 )

Records screened
(n =115 )

Records excluded
(n =70 )

Full-text articles assessed for eligibility
(n = 45 )

Full-text articles excluded, with reasons
(n =32 )

Studies included in qualitative synthesis
(n = 13 )

Studies included in quantitative synthesis (meta-analysis)
(n =13 )
